# The epidemiology of the blackleg pathogen, *Leptosphaeria maculans* , impacts fungicide resistance management strategies

**DOI:** 10.1002/ps.70866

**Published:** 2026-04-29

**Authors:** Alec J McCallum, Sean Roberts, Alexander Idnurm, Angela P Van de Wouw

**Affiliations:** ^1^ School of BioSciences, University of Melbourne Parkville Australia; ^2^ Syngenta Australia Charles Sturt University AgriPark Wagga Wagga Australia

**Keywords:** blackleg, canola, epidemiology, *ERG11*/*Cyp51*, infection timing

## Abstract

With the continual increase in detection of fungicide resistance in plant pathogens, there is an ever‐growing need for management strategies to reduce the chance of resistance evolving in the field, and if it does occur, provide strategies to ensure fungicides can still be used effectively. However, the development of effective management strategies requires a thorough understanding of the epidemiology of the pathogen and how this might impact on selection of fungicide‐resistant strains within a population. The blackleg pathogen, *Leptosphaeria maculans*, can infect canola (*Brassica napus*) throughout the growing season with yield loss associated with crown cankering or infection of the upper stems/branches. The disease cycle consists of infection of the plant early in the growing season, such as at the cotyledon up to the 4‐ to 6‐leaf stage, to induce cankering on cultivars that do not carry effective plant resistance (or *R*) genes. Infections later in the season lead to infections of the upper stems/branches. Hence, fungicides can be applied up to four times during the season to control disease symptoms. However, it is currently unknown whether fungicide applications late in the growth of the plant will lead to selection of resistance in the population, as the pathogen would require enough time to colonise the plant and undergo sexual reproduction. Here we show that infections as late as the 30% bloom stage do indeed have sufficient time for the pathogen to grow down and colonise the base of the stem which provides the substrate to persist for the following season and undergo sexual reproduction, i.e., the next generation of inoculum. This has implications for fungicide management strategies as it means any fungicide application throughout the growing season can lead to selection of resistance. © 2026 The Author(s). *Pest Management Science* published by John Wiley & Sons Ltd on behalf of Society of Chemical Industry.

## Graphical abstract

Understanding how infection timing drives disease is essential for the development of fungicide resistance management strategies. For blackleg disease of canola, all infection timings can contribute to the following generation, therefore all fungicide applications have the ability to select for resistance.

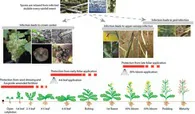

## 1 INTRODUCTION

Fungicides have become an integral part of disease control for plant pathogens across the world, with fungicide sales in 2019 totalling approximately $16.4 billion.^1^ The increased use of fungicides is driven by their economic benefits to growers, for example a single study on fluopyram‐amended seed treatment of soybean showed that the overall benefit of this application was $5.8 billion USD over 15 years.^2^ However, whilst the economic benefit of these fungicides drives their increased use, this leads to the emergence and an increase in the frequency of fungicide resistance. Fungicide resistance has now been identified towards 75% of the fungicide modes of action.^3^

The evolution of resistance or increased tolerance to fungicides results in the corresponding active ingredient having no or reduced efficacy in the field, leading to economic losses for farmers due to less disease control. In addition to the economic losses to farmers, the evolution of resistance leads to the reduced life span of the otherwise highly effective fungicides, causing economic losses also to the pesticide companies.^4^ To reduce the risk of resistance evolving, management strategies are essential to minimise the selection pressure imposed on the fungal population.^5^ There is general consensus that the evolution of fungicide resistance can be minimised by reducing the application of fungicides, rotation of fungicides with different actives or the application of mixtures of different chemical actives.^5^, ^6^ However, as more research is conducted on individual pathogens, it is becoming clear that universal strategies may not be appropriate and that specific strategies may be needed for individual pathogens and scenarios.^7^, ^8^, ^9^

Blackleg disease, caused by the pathogen *Leptosphaeria maculans*, is one of the major diseases of canola (oilseed rape, *Brassica napus*) worldwide,^10^ with fungicides being applied up to four times a season to help control this disease.^11^ The *L. maculans* disease cycle begins with the release of sexual ascospores from pseudothecia on infected stubble (crop debris) from the previous season (Fig. 1). The release of these ascospores occurs with each rainfall event during the canola growing season.^12^ When these ascospores land on cotyledons or true leaves, they germinate and invade the plant via stomata or wounds, and then colonise the intercellular spaces (Fig. 1). Lesions form within the following 3 weeks, whereby the fungus then produces asexual conidia that can lead to secondary infection via rain splash. The fungus then switches into a biotrophic phase as it grows down the petiole and into the stem before switching again into a necrotrophic phase later in the season as it invades and kills cells of the cortex, resulting in a blackened canker (Fig. 1).^13^ The necrotic stem canker restricts nutrient movement throughout the plant, leading to reduced yields and in severe situations the plants will lodge and die. Lastly, the pathogen then switches growth strategy again to an almost saprophytic stage whereby it remains within the crop debris. Here it undergoes sexual reproduction between canola growing seasons, giving rise to the sexual fruiting bodies and sexual spores to infect crops the following year.

**Figure 1:**
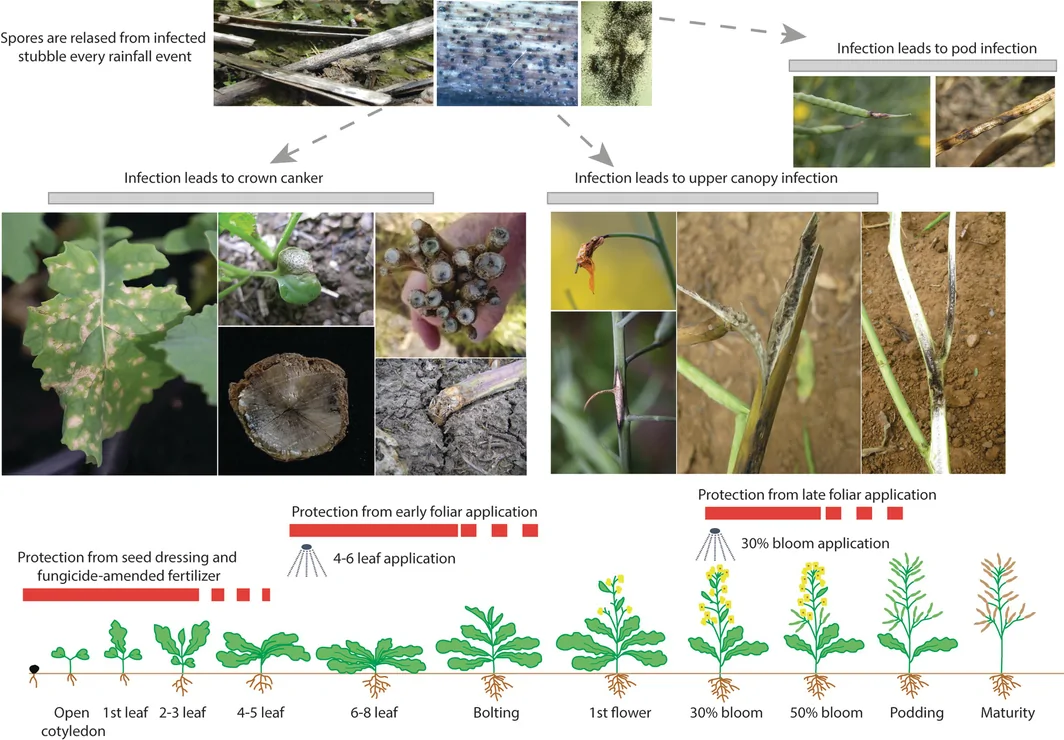
Disease epidemiology of blackleg disease of canola (*Brassica napus*), caused by *Leptosphaeria maculans*. Spores are released from infected stubble (crop debris) with each rainfall event during the growing season. If spores land on cotyledons and true leaves, the fungus grows down into the crown and causes severe crown cankers. However, if spores land on flowers, they infect and colonise them, leading to upper branch infection, termed upper canopy infection. Lastly, if spores land on pods, they will infect the pods and underlying seeds. Fungicides are used to control the various forms of blackleg disease, with seed‐dressings, fungicide‐amended fertilisers and early foliar applications designed to control crown canker, and 30% bloom applications designed to control upper canopy infection.

In addition to leaf infection, which leads to crown canker, the pathogen can also infect flowers and the upper branches, leading to symptoms termed upper canopy infection (Fig. 1) and is an issue in Australia and South Africa.^14^, ^15^ Upper canopy infection is a relatively new form of the disease as a consequence of earlier flowering times due to changed farming systems.^14^ In this scenario, ascospores are released during rainfall events and land on flowers/petals/branches. Whilst the specific strategy of entry into the plant is unknown, the fungus can colonise the petals, causing necrotic lesions on the flowers, before growing down the peduncle, leading to necrotic lesions within the branches. Lastly, infection events that occur during pod set lead to infection of the pods and the underlying seed (Fig. 1).^16^

To minimise the impact of blackleg disease, fungicides can be applied as seed dressing, as an amendment to fertilisers or as foliar sprays.^17^ The seed dressing and fungicide‐amended fungicides provide protection from the seedling stage through to around the 2‐ to 3‐leaf stage, whilst the foliar fungicides are generally used at the 2‐ to 4‐leaf stage and provide protection up until around the 6‐ to 8‐leaf stage of plant development (Fig. 1). In addition, fungicides can be applied at the 30% bloom (20 flowers open on the main stem) and 50% bloom growth stages (>20 flowers open on the main stem) to minimise the impact of upper canopy infection.^17^ These fungicide applications protect the flowers and early pod development from infection (Fig. 1) whilst avoiding high residual chemical levels after harvest. When controlling both crown canker and upper canopy infection using fungicides, up to four fungicide applications could be applied in a season, therefore placing strong selection pressure on the pathogen population for fungicide resistance. With fungicide resistance already having emerged to the demethylation inhibitor (DMI, Fungicide Resistance Action Committee [FRAC] Group 3) chemistries in Australia and Europe,^18^, ^19^, ^20^, ^21^ it is important to develop strategies to minimise the risk of resistance evolving to other active chemicals such as the succinate dehydrogenase inhibitors (FRAC Group 7) and quinone outside inhibitors (FRAC Group 11) fungicides.

To manage blackleg disease, it is necessary to understand the epidemiology of the pathogen with respect to both crown canker and upper canopy infection. It is currently unknown whether infections associated with upper canopy infection result in the pathogen being able to grow down into the crown, and then contribute to the population infecting the canola crop in the subsequent year. After harvesting it is common practice to leave the base of the canola plants, i.e., the stubble, for soil management reasons, although this material is the source of the inoculum in the following growing season. Understanding how timing of infection impacts on growth of the pathogen within the host is essential when making strategic recommendations for fungicide use. If the pathogen can grow from the upper infected tissue down into the crown (or lower parts of the plant), then it will persist in the stubble and be able to undergo sexual reproduction between seasons. If this is the case, then all fungicide applications, whether they are targeted at controlling crown canker or upper canopy infection, will contribute to selection of fungicide resistance. However, if the pathogen cannot grow from the infected upper infected tissue into the crown, then it will not persist in the stubble, nor be able to undergo sexual reproduction. Therefore, applications of fungicides for control of upper canopy infection will not contribute to the selection of fungicide resistance. As such, this study aims to investigate the epidemiology of the blackleg pathogen and relate this to sexual reproduction to determine appropriate recommendations for minimising the impacts of fungicide resistance.

## 2 METHODS

### 2.1 Production of *L. maculans* populations

To determine the impact of the timing of *L. maculans* infection on canola on its sexual reproduction, an inheritable gene with a population‐level marker was required. Resistance to the DMI fungicides fits this criterion as the insertion within the promoter of the *Cyp51* (also referred to as *ERG11*) gene is easily detectable using both PCR for tracking individual isolates and deep amplicon sequencing for analysis of population frequencies.^18^, ^19^

Four different types of population were developed, populations A through D (Table 1). Population A was fungicide sensitive (therefore lacking an insertion within the promoter of the *Cyp51* gene) and consisted of isolates with both MAT1‐1 and MAT1‐2 mating types. Population B was fungicide resistant (contained insertions within the promoter of the *Cyp51* gene) and consisted of isolates with both MAT1‐1 and MAT1‐2 mating types. Population C was fungicide sensitive but consisted of only MAT1‐1 isolates whilst population D was fungicide resistant and consisted of only MAT1‐2 isolates.

**Table 1:** *Leptosphaeria maculans* isolates used to develop populations used in this study

Population	Isolate	Year used	*Cyp51* allele*	Mating type allele
A	D2	2021, 2023	Susceptible	MAT1‐2
	D3	2021, 2023	Susceptible	MAT1‐1
	D16	2021	Susceptible	MAT1‐2
	D22	2021, 2023	Susceptible	MAT1‐1
	D23	2023	Susceptible	MAT1‐2
B	18BL153	2021	‐362:ins:5263	MAT1‐1
	20BL196	2021, 2023	−99:ins:237	MAT1‐1
	20BL198	2021	−132:com:296:16	MAT1‐2
	20BL201	2021, 2023	−414:ins:275	MAT1‐2
	18BL152	2023	−103:ins:275	MAT1‐2
	22BL128	2023	−116:com:296:16	MAT1‐1
C	D3	2023	Susceptible	MAT1‐1
	D22	2023	Susceptible	MAT1‐1
	IBCN306	2023	Susceptible	MAT1‐1
	IBCN309	2023	Susceptible	MAT1‐1
D	18BL152	2023	−103:ins:275	MAT1‐2
	20BL201	2023	−414:ins:275	MAT1‐2
	21BL210	2023	−99:ins:153	MAT1‐2
	21BL213	2023	−103:ins:275	MAT1‐2

* Notation indicates position relative to start codon: mutation type: size of insertion in base pairs.

*Note:* The *Cyp51* allele nomenclature refers to wild type (susceptible) or to the position and nature of the DNA insertions within the promoter region giving rise to reduced sensitivity to demethylation inhibitor fungicides.

Isolates were grown on 10% V8 agar at 22 °C with a 12 h:12 h photoperiod for 10 days, at which time pycnidiospores were collected by flooding plates with sterile water and filtering through sterile Miracloth. Pycnidiospores were counted on the haemocytometer and then adjusted to a concentration of 1 × 10^7^ spores/mL. For each population, spores from each isolate were mixed at equal proportions before aliquoting into 5‐mL tubes and stored at −20 °C until required for inoculations.

### 2.2 Infection timing experiments

A *Brassica napus* cultivar, 43C80, was used for all experiments as it is adapted for Australian growing conditions but has the ineffective major gene *Rlm3* (all isolates used in this study are virulent towards *Rlm3*) and low levels of quantitative resistance. Seeds were sown in polytubes (65 × 65 × 160 mm) containing Scotts Osmocote Premium Plus Superior Potting Mix (Mount Gambier, Australia). Experiments were designed so that each infection timing (treatment) consisted of five biological replicates, with each replicate containing 20 individual plants. Biological replicates were fully randomised across the experiment using trial design software, DiGGer. All biological replicates were inoculated at the same times throughout the experiment.

All experiments were sown in April and grown in a polyhouse with no controlled conditions so that plants grew in an environment similar to field conditions. Since these experiments were conducted over the Australian canola growing season (April to November), the polyhouse remained only 3–5 °C warmer than outside conditions. The polyhouse was not fully enclosed, with the walls open, therefore humidity did not differ from outside conditions. As such, the conditions in which the experiment were conducted reflect on‐farm conditions. Furthermore, no plants were placed under high humidity following inoculation. To minimise the risk of external inoculum interfering with the experiments, the experiments were initiated in early April prior to the initiation of blackleg spore release in the field (which typically begins mid‐late June). In addition, throughout the course of the experiment susceptible trap plants (seedlings) were placed within the polyhouse and assessed for disease to test for the presence of external inoculum. Over the course of the season, lesions were detected on seedling plants twice, both times in August, and lesion numbers were low, suggesting external inoculum is unlikely to be contributing to the experiments. The dates associated with the experimental activities are listed in Supporting Information, Table S1.

In 2021 and 2023, all seedlings at the open cotyledon stage were inoculated with population A (Fig. 2). Cotyledons were wounded with a needle and then pycnidiospores suspended in water (1 × 10^6^ spores/mL) were sprayed on the wounded plants using a Preval hand‐held spray gun. Plants were sprayed until liquid run‐off to assure complete coverage. For the cotyledon–cotyledon timing of infection treatment, an hour after inoculation with population A, the cotyledons were re‐wounded and sprayed with population B (Fig. 2). Lesion development was scored on all cotyledons 2 weeks following inoculation to confirm inoculations were successful (data not shown).

**Figure 2:**
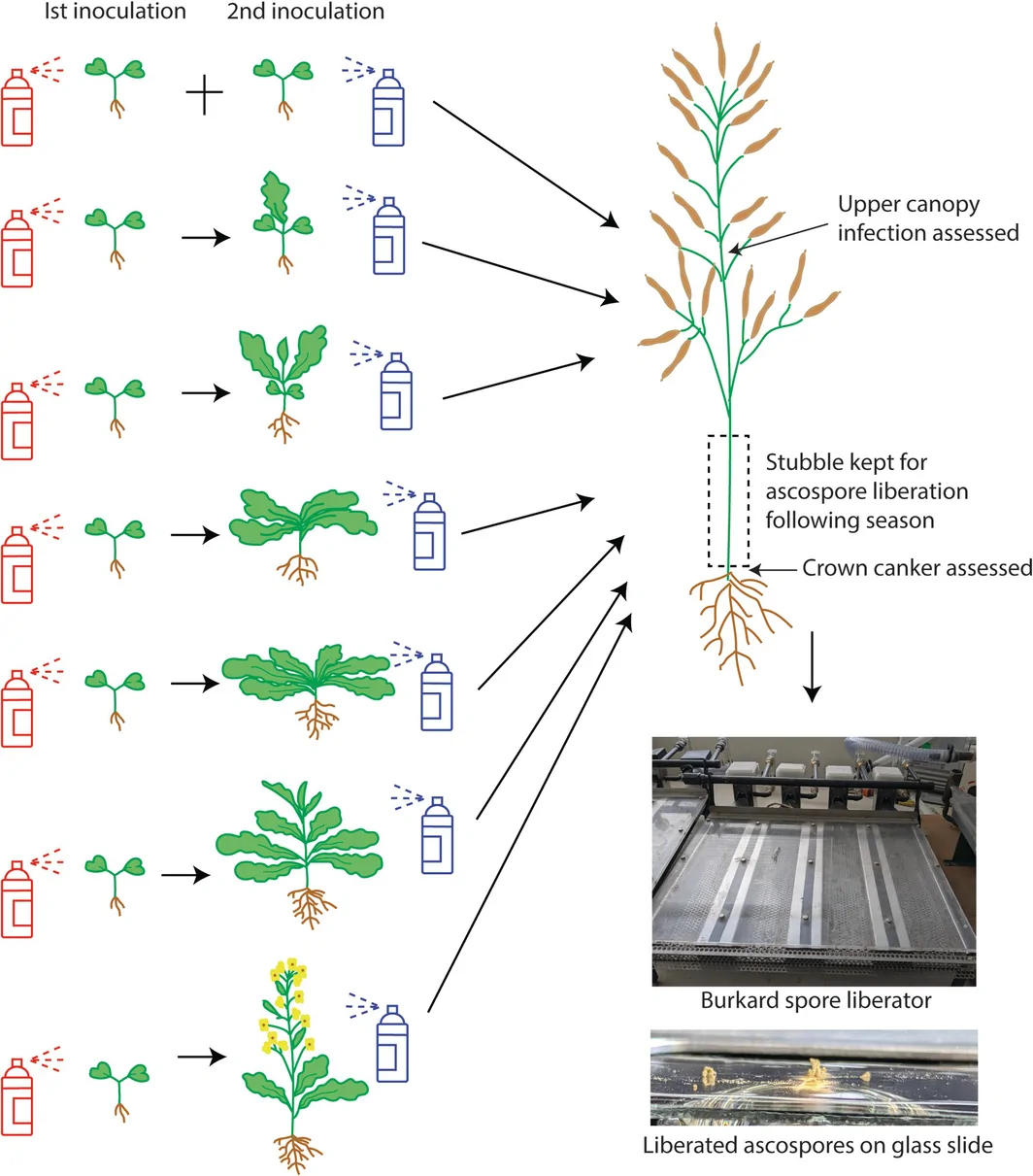
Methodology for testing the impact of infection timing on disease progression and sexual reproduction. All plants were inoculated at the cotyledon stage with population A (fungicide‐sensitive population). Following this, plants were then reinoculated at various growth stages with population B (fungicide‐resistant population). All plants were allowed to grow to physiological maturity before being assessed for crown canker development and upper canopy infection development (30% bloom infection timing). Stubble was kept from all plants and used for ascospore liberation (using a Burkard spore liberator) the following season to determine the impact of infection timing on sexual reproduction.

For the remaining timings of infection, the leaves were wounded and sprayed with population B at the 1‐, 2‐, 4‐, 6‐ and 8‐leaf growth stages. For the 30% bloom inoculation timing, plants were wounded at the junction of the third branch by light scraping with a sterile scalpel blade before being sprayed with the population B spores. This provided a surface for the spore solution to rest and allow spores to germinate and infect. It did not provide an entry point for the fungus, therefore preventing unnatural infection events.

In 2023, a smaller experiment was also conducted whereby only two infection timings were tested, cotyledon–cotyledon and cotyledon–30% bloom using populations C and D. In this experiment, all seedlings were inoculated at the open cotyledon stage with population C and then the follow‐up inoculations (cotyledon or 30% bloom) were inoculated with population D, using the same procedures as described above.

Disease severity was assessed at physiological maturity for both crown canker and upper canopy infection. For crown canker severity, plants were cut at the crown of the plant and the cross‐sectional internal infection was measured.^22^ For upper canopy infection, the length of the external lesion was measured (millimetres) from the site of inoculation at the branch junction. Significant differences in crown canker severity were determined using analysis of variance. Data were square root transformed prior to analysis with GenStat. For each treatment, 20 plants (technical replicates) were averaged to provide a single biological replicate. Five biological replicates were then analysed as an experimental unit for each treatment.

Following disease assessment, 40–50 cm of the stem of the plant (from the base) was kept as stubble. All 20 stems from each replicate were placed in mesh bags and placed on the ground in a field over the summer to allow natural maturation of pseudothecia and the production of ascospores following sexual reproduction. Each year, the same field was used for stubble maturation. Maturation occurred naturally from November (when the stubble is harvested) through to the following July. Stubble was inspected under the microscope for the presence of mature pseudothecia before spores were liberated.

### 2.3 Population tracking using deep amplicon sequencing

Ascospores were collected from mature stubble in July the year following the initial experiment using a Burkard Spore Liberator (manufactured Brisbane, Australia) as previously described.^23^ A Burkard Spore liberator consists of four individual chambers with a funnel at the end with a small aperture (Fig. 2). Behind the aperture is a holder where glass slides can be placed. Stubble is placed within the chamber, with air being sucked through the chamber. When spores are released from the stubble, they are sucked through the aperture and attach to the glass slide. For each biological replicate, the 20 stems were placed in a chamber of the liberator and ascospores liberated for 2 h onto glass slides. The ascospores captured on the glass slides were transferred into 10% liquid V8 and allowed to grow for 48 h and then freeze dried as previously described.^18^ Genomic DNA was extracted using the Qiagen DNeasy Plant Mini Extraction kit (manufactured Hiden, Germany) as per manufacturer's instructions.

The promoter region of the *Cyp51* gene was amplified from samples using the primers and conditions previously described^18^ with amplicons checked using gel electrophoresis. Products were sequenced using the Australian Genome Research Facility (Melbourne, Australia), processed with NovaSeq control software (v1.3.1.59007) and real‐time analysis (v4.29.3). The Illumina DRAGEN BCL Convert 07.031.732.4.3.6 pipeline was used to generate the sequence data. More than 4 000 000 reads were yielded from each sample. Sequence reads were analysed using a similar pipeline to Scanlan *et al*.^18^ Briefly, sequence reads were trimmed and filtered using *bbduk.sh* in BBMAP v39.01 to quality >35 and minimum length >100 with both the raw reads and filtered reads verified using FastQC v0.11.9. To estimate allele frequencies, the sequencing reads were compared to an index of the known alleles used in the experiment (Table 1) in *kallisto* v0.48.0. The resulting transcripts per million values were converted to allele frequency by dividing by 10 000.

### 2.4 Tracking *in planta* growth of *L. maculans* using PCR

To determine the extent of growth of the pathogen when inoculated at the 30% bloom stage, stem samples were collected at physiological maturity for eight replicate plants (four plants inoculated with populations A and B, and four plants inoculated with populations C and D). Samples 1 cm in length were taken at the crown as well as the site of the 30% bloom inoculation. Two additional samples were taken at approximately one‐third (referred as growth point 2) and two‐thirds (referred as growth point 3) of the distance between the crown and 30% bloom inoculation point. Samples were freeze dried before DNA was extracted using the Qiagen DNeasy Plant Mini Extraction kit as per manufacturer's instructions.

To detect fungal growth in each of the stem samples, two different PCRs were performed: *Cyp51* promoter region (EPS1F 5′‐AGCACCCATGGACCACGG‐3′; EPS6R 5′‐CAGGATAAAGGAGGCGAAG‐3′)^19^ and the mating type (MAT1‐1 5′‐TGGCGAATTAAGGGATTGCTG‐3′; MAT1‐2 5′‐CTCGATGCAATGTACTTGGAGC‐3′; MAT 5′‐CGGAGGTGAAGTTGAAGCCG‐3′)^24^ using conditions previously reported. For stem samples from plants inoculated with populations A and B, only the *Cyp51* promoter region was used as each population consisted of isolates of both mating types. The presence of resistant isolates in the stem samples was indicted by insertions in the promoter region, whilst the presence of the susceptible population was indicated by a lack of insertions. For stem samples from plants inoculated with populations C and D, both the *Cyp51* promoter and *MAT* PCRs were performed. The lack of insertions in the *Cyp51* promoter and the presence of the *MAT1‐1* allele were indicative of population C whilst insertions within the *Cyp51* promoter and the presence of *MAT1‐2* allele were indicative of population D. A subset of samples was repeated to rule out false positives, with 100% correlation between PCRs detected (data not shown).

## 3 RESULTS

### 3.1 Impact of infection timing on disease progression

Populations of isolates were inoculated onto plants at differing growth stages and disease development assessed. Population A, i.e., a fungicide‐sensitive population with both mating types, was inoculated onto cotyledons of all plants at the start of each experiment in both 2021 and 2023, and as expected virulent reactions were detected on cotyledons of all plants (data not shown). Population B, i.e., a fungicide‐resistant population with both mating types, was then inoculated at varying growth stages from cotyledon through until the 8‐leaf stage and at 30% bloom (Fig. 2). At maturity, there was no significant difference in crown canker severity for any of the infection timings, except for the 2021 cotyledon–cotyledon infection timing (Fig. 3). For the infection timing of 30% bloom, stem lesion development at the site of inoculation was also measured at physiological maturity. In 2021, the average branch lesion length was 32 mm and in 2023 the average branch lesion length was 17 mm.

**Figure 3:**
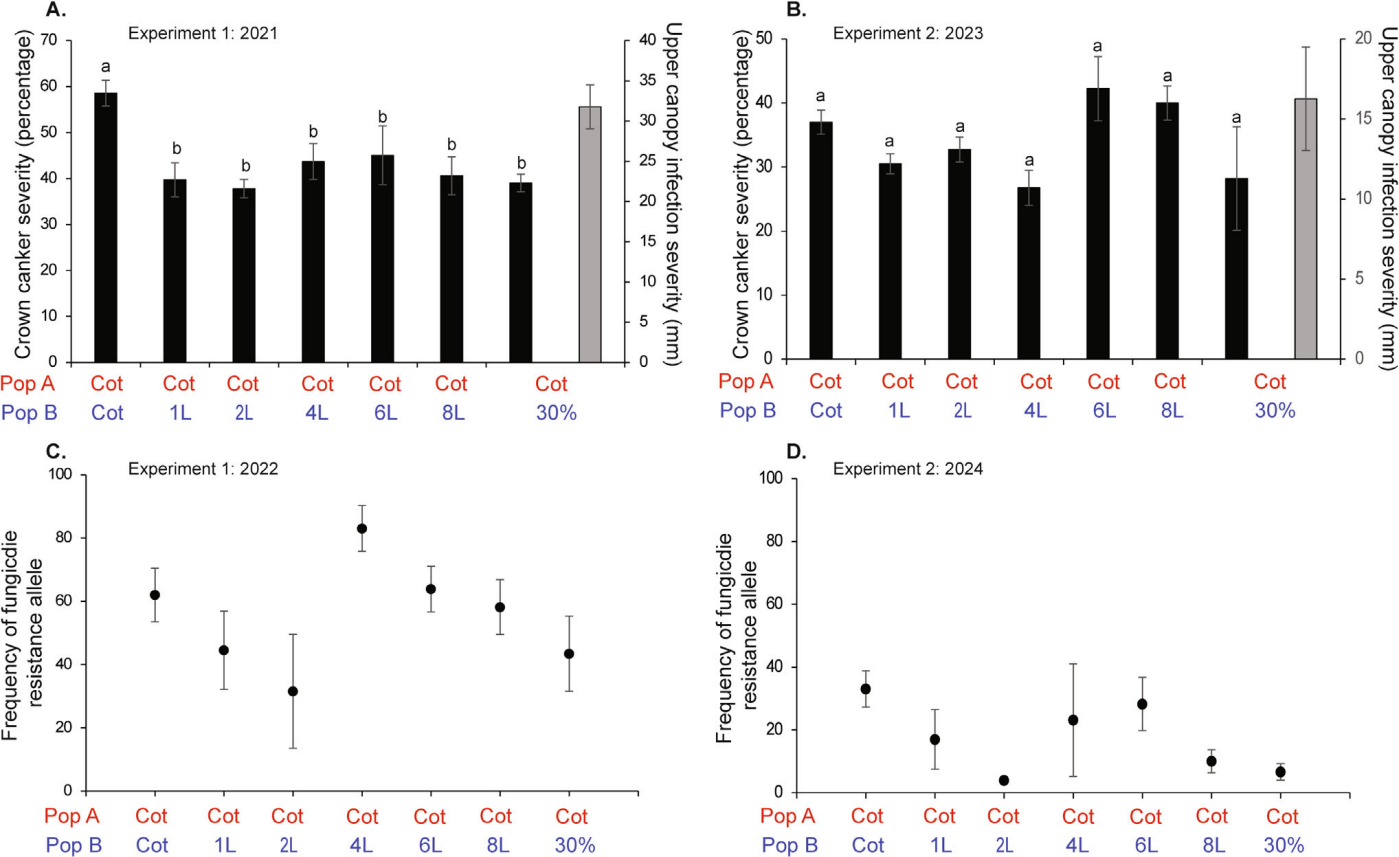
Disease severity and allele frequency data from two experiments investigating the impact of different inoculation timings of *Leptosphaeria maculans*. Experiment 1 was inoculated in 2021 and then allele frequencies determined in 2022 following sexual reproduction, whilst experiment 2 was inoculated in 2023 and allele frequencies assessed in 2024. Plants were inoculated with two different populations (Pop A and B) at different timings. Cot, cotyledon; 1L, 1‐leaf; 2L, 2‐leaf; 4L, 4‐leaf; 6L, 6‐leaf; 8L, 8‐leaf; 30%, 30% bloom. (A) and (B) Crown canker severity was measured for all inoculation timings (black bars) and upper canopy infection was measured for the 30% bloom inoculation timing (grey bars). (C) and (D) Following sexual reproduction, the frequency of the *Cyp51* resistance allele was determined in the subsequent population. Data are averages of five biological replicates, with 20 individual plants per rep. Statistical differences are indicated by a different letter (*P* < 0.001). Error bars represent standard errors.

### 3.2 Impact of infection timing on sexual reproduction

To determine whether the timing of late infections during the growing season impacts the ability of the pathogen to undergo sexual reproduction to contribute to the next generation, stubble was collected from experiments, matured, and then the ascospores were liberated and the frequency of fungicide resistance determined. For both years of the experiment and all infection timings the fungicide‐resistant allele was detectable following sexual reproduction, suggesting that regardless of when infection occurs, isolates of population B had enough time to grow down the stem and into the crown to undergo sexual reproduction in the remnant stubble. Interestingly, in 2021, the fungicide‐resistant allele was detectable between 0.061% and 98.3% with a mean of 53.6% across the experiment, however it was much lower in the 2023 experiment, detectable between 2.87% and 100% with a mean of 24.0% (Fig. 3). In both experiments, a trend was detected whereby the frequency of the fungicide‐resistant allele decreased between the cotyledon–cotyledon and cotyledon–2‐leaf timings and then increased again at the cotyledon–4‐leaf infection timing stage (Fig. 3).

### 3.3 Fungal growth associated with upper canopy infection

Whilst the fungicide resistant allele was detected from stubble collected from the 30% bloom inoculation strategy (upper canopy infection), it was possible that the fungicide‐resistant population was undergoing sexual reproduction in the upper part of the stubble whilst the fungicide‐sensitive population was undergoing sexual reproduction independently at the crown. To determine whether the two populations were indeed meeting and undergoing sexual reproduction, stem sections were collected at maturity and growth from each of the populations determined by PCR (Fig. 4). As expected, population A was detected at the crown in all samples and population B was detected at the 30% bloom inoculation point for all samples. For population B, growth was detected at growth point 2 and 3 in all plants sampled and at the crown in two out of the four plants (Fig. 4). For population A, growth was detected at growth points 2 and 3 for three of the four plants sampled but never detected at the 30% bloom inoculation point. These results suggest that the isolates are indeed meeting within the stubble to allow sexual reproduction to occur.

**Figure 4:**
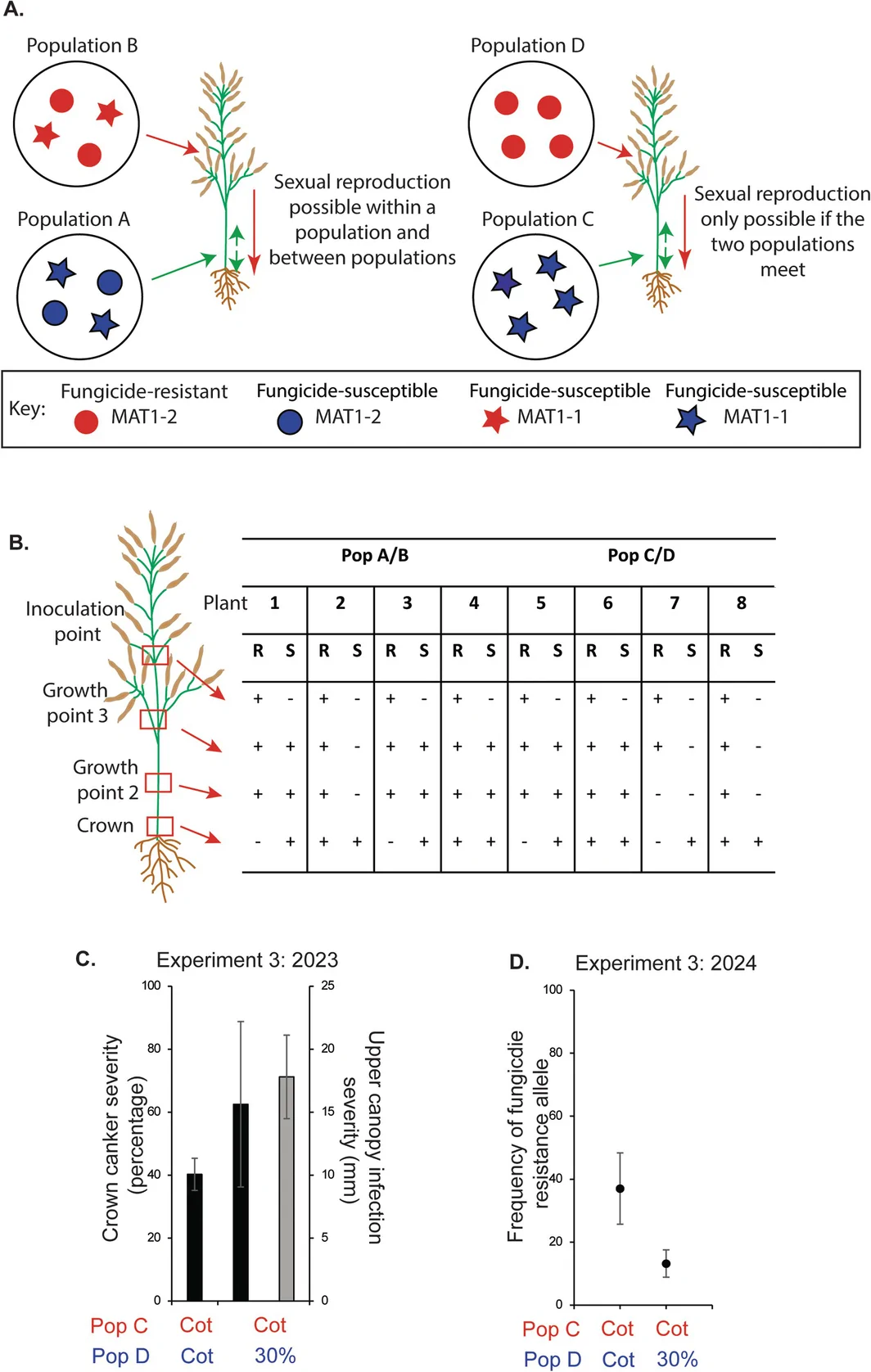
Growth of isolates following inoculation of *Leptosphaeria maculans* at the 30% bloom growth stage was determined using PCR of the *Cyp51* gene. (A) Schematic of the different populations used for inoculations. Populations A and B can undergo sexual reproduction without interacting with each other since both populations consist of isolates with both mating types. Populations C and D can only undergo sexual reproduction if the two populations meet as each population only has isolates from a single mating type. (B) Growth of the fungicide‐susceptible (S) or fungicide‐resistant (R) isolates was determined using PCR (+ indicates detection, − indicates absence of detection) at four different locations within the main stem of the plant. In addition to experiments using populations A and B (see Fig. 3), an additional experiment was carried out using populations C and D, whereby single mating types were present in each of the populations. (C) Disease severity data for both crown canker and upper canopy infection when populations C and D were inoculated at either the cotyledon (Cot) or 30% bloom (30%) growth stages. (D) Following sexual reproduction, the frequency of the *Cyp51* resistance allele was determined in the subsequent population.

To further validate the growth of isolates from the upper canopy infection inoculation point, a separate experiment was conducted with two different populations. The first consisted of a fungicide‐sensitive population containing only MAT1‐1 isolates (population C) and the second consisted of a fungicide‐resistant population containing only MAT1‐2 (population D). Therefore, to undergo sexual reproduction, the two populations must meet within the plant. To determine the extent of fungal growth of the isolates inoculated at the 30% bloom growth stage, sections of stem were collected and fungal growth detected via PCR (Fig. 4). As expected, population C (fungicide‐sensitive) was detected at the crown in all plants sampled, whilst population D (fungicide‐resistant) was detected at the 30% inoculation point in all plants sampled (Fig. 4). Population D was detected at growth point 3 in all plants sampled, at growth point 2 in three of four plants, and at the crown in two of the four plants sampled. Population C was detected at growth points 2 and 3 in two of the four plants sampled and never at the 30% bloom inoculation point.

When ascospores were liberated from stubble collected from the experiments using populations C and D, the fungicide‐resistant allele was detected at a frequency of 13.2%, confirming that the two populations were indeed meeting and undergoing sexual reproduction (Fig. 4). As a control, populations C and D were also both inoculated at the cotyledon stage with the resistant allele being detected at 37.0% following sexual reproduction.

## 4 DISCUSSION

Minimising the risk of the spread of fungicide resistance is essential for maintaining the longevity and efficacy of fungicides to protect crops. Whilst there are general recommendations for the management of fungicide resistance, these are usually based around hypothetical control strategies and may not always be applicable to all scenarios, therefore it is essential to have a thorough understanding of the pathogen/disease being managed. In the current study we have investigated the epidemiology of blackleg disease, a global problem that impacts oilseed production, to determine the impact of infection timing on disease progression, sexual reproduction and its impact for causing disease in the subsequent growing season. This is particularly important with the increase in reports of blackleg symptoms developing late in the growing season, termed upper canopy infection, in places like Australia and South Africa that impact yield and therefore are targeted by late‐season applications of fungicides.^14^, ^15^ In addition to fungicides, another control measure against diseases is plant resistance. Like fungicides, extensive use of the same resistance genes places strong selective pressure onto pathogen populations. Hence, this current research may also provide insights into the sustainable management of plant resistance genes.

We have shown that infection at all growth stages between cotyledon and 30% bloom (20 open flowers on the main stem) can lead to disease, causing either crown canker or upper canopy infection, and more importantly the pathogen can undergo sexual reproduction within the remaining stubble. Analysis of fungal growth with PCR showed that not only could the fungicide‐resistant population grow down towards the crown, but the fungicide‐sensitive population could also grow up away from the crown, being detected as high as growth point 3 (Fig. 4). This is consistent with a previous study, whereby growth of fluorescently labelled *L. maculans* isolates was observed both towards the crown and towards the upper stems.^25^ The authors at the time suggested that their observation may have been an artifact of the growth conditions, however the similar observation detected in the current study suggests that this observation may be accurate, although a biological explanation as to why the pathogen would grow up instead of down remains unknown.

The ability of the pathogen to grow down the stem and into the crown, irrespective of infection timing, means that any selection pressure imposed on the pathogen throughout the growing season will contribute to evolution of resistance as the pathogen can undergo sexual reproduction and pass on its genes. It has been suggested that for some diseases, such as Asian soybean rust, delay of infection can contribute to the management of fungicide resistance.^26^ For Asian soybean rust, this delay reduced the length of time the fungus was exposed to the fungicides and therefore decreased disease pressure. However, based on the findings of our current study, such strategies are unlikely to be useful for management of fungicide resistance in the blackleg pathogen since infection at any growth stage still allows enough time for the pathogen to colonise the plant to enable it to contribute to the population in the following growing season. However, timing of infection may still be important in the management of the blackleg pathogen. In both years of the current study, an increase in the frequency of fungicide‐resistant populations was detected at the 4‐leaf inoculation timing (Fig. 3), suggesting that this growth stage may be more sensitive to inoculation than other timings. A historic study looking at the effect of plant age on blackleg infection found that the severity of stem lesions was highest in plants inoculated at 3‐leaf (score of 4.1) followed by 1‐leaf (3.9), 6‐leaf (3.0), flower bud (2.0) and open flower stages (0.8).^27^ This is partly consistent with our study, but why plant age might affect disease susceptibility is unknown. In our study we did not find an increase in disease severity at the 4‐leaf inoculation time point, but we did see an increase in the frequency of resistant isolates the following generation, suggesting that they could colonise more rapidly when inoculated at the 4‐leaf growth stage compared to other stages. Leaf age has been shown to significantly impact disease development in other plant–host interactions such as the susceptibility of strawberry to *Mycosphaerella fragariae*, Arabidopsis infection by *Pseudomonas syringae*, and infection of wheat by *Puccinia striiformis*.^28^, ^29^, ^30^ Similarly we did see an increase in disease severity for the 2021 experiment when the fungicide‐resistant population was inoculated at the cotyledon stage. This may suggest that there is increased susceptibility when inoculated early, but this was not consistent in the 2022 experiment. The specific reason for differences in leaf susceptibility remains unknown, with options including higher nutrition in younger leaves, anatomical structures providing barriers and varying levels of basal defence signalling.^30^, ^31^ One potential limitation of the current study is that a single cultivar was used for all experiments, and this cultivar has low levels of quantitative resistance. If these same experiments were conducted in a cultivar with higher levels of quantitative resistance, this presumably would cause some restriction of fungal colonisation within the plant and therefore could reduce the impact of the later infections on sexual reproduction of the pathogen. Recent studies looking at the impact of both host genetics and fungicides to control blackleg have shown that genetic resistance reduces disease more reliably than fungicides,^32^ suggesting that the quantitative resistance could impact on the evolution of the pathogen and should be considered in future experiments.

The ability of all infection timings to contribute to the following generation via sexual reproduction is an essential factor when developing strategies for minimising fungicide resistance. First, this finding suggests that applications of a fungicide at any growth stage will place selection pressure on the population and potentially select for fungicide‐resistant isolates. Equally, however, this information can potentially provide a strategy for minimising fungicide resistance. Previous studies have shown that severity of crown canker disease decreases post the 5‐leaf stage: disease is still present but the severity of the crown canker does not cause any significant yield loss.^33^ In the current study, we show that infection events post the 5‐leaf stage still provide enough time for the pathogen to colonise the stem and contribute to the following generation through sexual reproduction, meaning that fungicide applications within this window are likely to be increasing selection for fungicide resistance, without providing any yield benefits. Therefore, a potential management strategy for reducing the risk of fungicide resistance could be to allow infections to occur between the 5‐leaf and 30% bloom growth stages, allowing susceptible individuals present in the population to infect the plant, grow into the stem and undergo sexual reproduction, thereby diluting the frequency of any resistant isolates that have been selected for using fungicides at the seedling stage. Whilst the fungicide‐susceptible isolates could cause some disease, this is unlikely to impact yield loss but would likely balance the proportion of resistant isolates in subsequent generations. If this hypothesis were tested and thus the approach did indeed slow selection of fungicide resistance, then it would be advisable to avoid spraying fungicides at the early foliar timing (4–6‐leaf stage of development) to allow fungicide susceptible isolates to infect.

Whilst various strategies to prevent fungicide resistance are well documented, recent studies have shown that the effectiveness of these strategies can be influenced by the presence of resistant isolates already in the population.^7^ In many regions around the world, resistance to the DMI fungicides have already been detected in *L. maculans* populations,^20^, ^21^, ^34^ suggesting that the application of management strategies to prevent fungicide resistance may not be effective. However, a recent study in *Pyrenophora teres* f. *teres* has shown that the frequency of resistance isolates can be reduced through inoculation with susceptible individuals.^35^ Therefore, for *L. maculans* populations whereby resistance has already been detected, limiting the use of fungicides to only early seedling protection, so that susceptible fungicides can then infect and proliferate to the next generation, has the potential to reduce resistance allele frequencies and improve the longevity of the DMI fungicides.

Whist not all regions apply fungicides to control blackleg disease, the use of fungicides at the 10–50% bloom stage for the control of Sclerotinia stem rot, caused by *Sclerotinia sclerotiorum*, is common.^10^ Since the blackleg pathogen has enough time to grow into the stem and sexually reproduce even when infection occurs as late as 30% bloom, it suggests that applications aimed at controlling Sclerotinia stem rot have the inadvertent potential to select for fungicide resistance in the blackleg pathogen. The proportion of applications targeted for Sclerotinia stem rot control in Australian crops is currently unknown, however in the UK, 24–35% of fungicide applications are targeted towards Sclerotinia stem rot, whilst only 10–29% are targeted for blackleg, and these blackleg‐targeted applications are all at the seedling stage.^34^, ^36^ As in Australia, a high proportion of DMI fungicide resistance has been detected in the UK.^20^, ^34^ One possible explanation for the high level of DMI fungicide resistance could be the use of DMI fungicides at the 30% bloom timing.

In the current study, no fungicides were used in the experiments and therefore there was no selection pressure applied to the fungicide‐resistant populations. When both populations were inoculated at the cotyledon stage, the starting frequency of the fungicide resistance allele of *Cyp51* was 50%, and following one generation with no selection pressure, the frequency of the resistance allele was between 25.0% and 90.9%. These results suggest that there is minimal fitness penalty associated with the DMI fungicide resistance, but the variation in frequency across experiments was large. The reason for this variation remains unknown at this stage. Whilst the data suggest no or low fitness penalty, the current study only looks at the impact of a single season with no selection. Further work would be needed to analyse long‐term changes following multiple seasons of no fungicide selection. If long‐term no or low fitness penalty was associated with DMI‐fungicide resistance this could make management of resistance in the field difficult as it may persist even in the absence of the fungicide.

## 5 CONCLUSIONS

The development of management strategies to prevent or minimise the rise of fungicide resistance is essential. For blackleg disease of canola, there is the possibility to use the epidemiology of the pathogen as a tool to minimise the selection of resistance since infection of susceptible isolates at any growth stage can contribute to the following generation. Understanding the dynamics of the pathogen population and how disease epidemiology influences potential selection is essential when trying to manage disease as well as fungicide resistance.

## CONFLICT OF INTEREST

The authors declare no conflict of interest.

## Supporting information


**Table S1.** Experimental dates.

## Data Availability

The data that support the findings of this study are available on request from the corresponding author. The data are not publicly available due to privacy or ethical restrictions.
