# Supplementary material for: The epidemiology of the blackleg pathogen, Leptosphaeria maculans , impacts fungicide resistance management strategies
Source: Pest Manag Sci. 2026 Apr 29;82(11):10786–95. doi: 10.1002/ps.70866 (PMC13665614; doi:10.1002/ps.70866)
Supplement: Supplementary file 1 [file PS-82-10786-s001.docx]

**Supplementary table 1** Experimental dates

| **Activity** | **Experiment 1 - 2021** | **Experiment 2 - 2023** | **Experiment 3 - 2023** |
| --- | --- | --- | --- |
| Sowing | 24/4/2021 | 30/4/2023 | 30/4/2023 |
| Cotyledon inoculations | 7/5/2021 | 16/5/2023 | 16/5/2023 |
| 1^st^ leaf inoculations | 11/5/2021 | 24/5/2023 | N/A |
| 2^nd^ leaf inoculations | 13/05/2021 | 30/5/2023 | N/A |
| 3-4 leaf inoculations | 18/5/2021 | 06/06/2023 | N/A |
| 5-6 leaf inoculations | 25/05/2021 | 20/06/2023 | N/A |
| 6-8 leaf inoculations | 9/06/2021 | 04/07/2023 | N/A |
| 30% bloom | 7/7/2021 | 31/07/2023 | 31/7/2023 |
| Crown canker assessment | 05/10/2021 | 11/10/2023 | 11/10/2023 |
| UCI assessment | 05/10/2021 | 11/10/2023 | 11/10/2023 |
| Spore liberation | 10/07/2022 | 15/07/2024 | 15/07/2024 |
